# Floridoside production by the red microalga *Galdieria sulphuraria* under different conditions of growth and osmotic stress

**DOI:** 10.1186/s13568-016-0244-6

**Published:** 2016-09-13

**Authors:** Marta Martinez-Garcia, Marc J. E. C. van der Maarel

**Affiliations:** Aquatic Biotechnology and Bioproduct Engineering, Engineering and Technology Institute Groningen (ENTEG), University of Groningen, Groningen, The Netherlands

**Keywords:** Red microalgae, Compatible solute, Osmotic stress, Floridoside, Galactosylglycerol, *Galdieria sulphuraria*

## Abstract

Floridoside is a compatible solute synthesized by red algae that has attracted considerable attention due to its promising antifouling and therapeutic properties. However, research on industrial applications of floridoside is hampered by limited compound availability and the development of a production process yielding high amounts of this glycoside has not been explored yet. In the present work, floridoside accumulation by the red microalgae *Galdieria sulphuraria* under different conditions was investigated in order to optimize the production of this glycoside in this microalgae. *G. sulphuraria* shows consider advantages over other red algae as potential industrial producer of floridoside due to its unicellular nature, its ability to grow heterotrophically in complete darkness and its acidophilic lifestyle. The main compatible solute accumulated by *G. sulphuraria* under salt stress was purified, identified as floridoside by ^1^H-NMR and used as standard for quantification. Our results showed that applying the osmotic stress after the cells had grown first in medium with no salt resulted in higher floridoside yields compared to those obtained in cells growing under osmotic stress from the beginning. Among several parameters tested, the use of glycerol as carbon source for cell growth showed the most significant impact on floridoside accumulation, which reached a maximum of 56.8 mg/g dry biomass.

## Introduction

Compatible solutes are small organic molecules synthesized by cells under various stress conditions that can be accumulated at high intracellular concentrations without interfering with the normal functioning of the metabolism (da Costa et al. 1998; Roberts 2005; Hagemann and Pade 2015). Floridoside (2-*O*-α-d-galactopyranosylglycerol) is a compatible solute synthesized by almost all red algae species, except the members of the class *Ceramidiales*, under high osmotic pressure conditions (Kirst and Bisson 1979; Reed 1985; Ekman et al. 1995). This glycoside also constitutes the major soluble pool of carbon fixed by photosynthesis and is a precursor for cell wall polysaccharides in some species (Li et al. 2001, 2002). Apart from its in vivo role as osmolyte, floridoside has been described to have certain properties that have raised the interest in this molecule. Hellio et al. (2004) reported that floridoside is able to inhibit the settlement of cryptid larvae on the surface of underwater devices, suggesting its application as non-toxic, natural compound for preventing biofouling, a worldwide problem estimated to cause a loss of billions of dollars to the marine industry (Callow and Callow 2002). Moreover, floridoside is a potential therapeutic agent with the ability to modulate the immune response (Courtois et al. 2008; Kim et al. 2013) and to promote bone formation (Ryu et al. 2015). Floridoside shares structural similarity with 2-*O*-α-d-glucopyranosylglycerol (GG), a compatible solute accumulated by cyanobacteria that is considered a promising moisturizing agent (Thiem et al. 1997), a non-cariogenic, low calorie sweetener (Takenaka and Uchiyama 2000) and a protein stabilizer (Sawangwan et al. 2010). The structural similarity suggests that floridoside might also be functional in these applications.

The development of industrial applications of floridoside is hampered by limited compound availability. Chemical synthesis of floridoside has been reported, albeit with insufficient yields and requiring a sequence of steps to direct the reaction towards the stereochemically pure product (Weïwer and Linhardt 2008). To date, there are no studies describing the enzymatic production of floridoside, but this strategy has been used for the synthesis of the related compounds 3-*O*-β-d-galactopyranosylglycerol and GG (Takenaka and Uchiyama 2000; Goedl et al. 2008; Wei et al. 2013; Jeong et al. 2014). Although the use of glycosidases takes advantage of the stereospecificity of these enzymes when forming a linkage in a single step, it suffers from a lack of regioselectivity towards a specific hydroxyl group, leading to the product being a mixture of regioisomers (Scigelova et al. 1999) that can complicate the downstream processing. Extraction of floridoside from the natural producers, i.e. red algae, is a promising alternative but requires the optimization of the cultivation conditions to increase the production of this glycoside by the cells.

In the present study, we opt for the extremophilic red microalgae *Galdieria sulphuraria* as floridoside producer. This unicellular rhodophyta is one of the most primitive eukaryotes on earth (Yoon et al. 2006) and thrives in acidic environments with pH values from 0 to 4 and temperatures up to 56 °C. *G. sulphuraria* is also a metabolically flexible species, being able to grow in complete darkness using a wide range of carbon sources (Gross and Schnarrenberger 1995) and displaying tolerance to various stresses (Schönknecht et al. 2013; Minoda et al. 2015; Pade et al. 2015). Its unicellular nature would confer *G. sulphuraria* an advantage over other multicellular red algae species for large scale cultivation and would allow to avoid seasonal variations in floridoside production reported for seaweed harvested from marine habitats (Kasrten et al. 1993; Meng and Srivastava 1993; Kerjean et al. 2007). Moreover, its acidophilic lifestyle would considerably reduce the risk of microbial contamination during large-scale fermentations.

In this work, we analyse floridoside accumulation under different cell cultivation and osmotic stress conditions in order to optimize the production of this glycoside in *G. sulphuraria*.

## Materials and methods

### Strain and cultivation conditions

*Galdieria sulphuraria* strain SAG 108.79 was purchased from the culture collection of the University of Göttingen (Sammlug von Algenkulturen, Göttingen, Germany). Cells were maintained growing on plates of Allen’s mineral medium (Allen 1959) at pH 4 with 1.5 % (w/v) agar at 40 °C and constant illumination of 100 µE/m^2^s. Colonies were transferred to a fresh plate once a month. For liquid cultures, *G. sulphuraria* was grown at 40 °C in complete darkness on a rotary shaker at 150 rpm in Allen medium at pH 2 supplemented with 1 % (w/v) glycerol and, when applicable, NaCl at a concentration of 0.5, 1 or 1.5 M. Cell growth was monitored by measuring the OD at 800 nm.

In order to purify floridoside that could be used as standard, cells were grown until late exponential phase and then salt-stressed with 1 M NaCl for 24 h at 40 °C. To construct the time-course of floridoside and glycogen content after salt addition, cells were grown until late exponential phase and then salt-stressed with 1 M NaCl for 0, 4, 8, 16, 24 and 48 h at 40 °C. To determine the effect of the carbon source and type and concentration of the osmotic agent on floridoside production, cells were grown on 1 % (w/v) carbon source (glycerol, galactose or glucose) until late exponential phase, harvested and washed with ultra-pure water and resuspended in 100 mL of the osmotic agent (NaCl, KCl, CaCl_2_ or sorbitol) at different concentrations (when applicable, 0.5, 1 or 1.5 M) for 24 h at 40 °C. To test the effect of temperature on floridoside production, cells were grown until late exponential phase on 1 % glycerol, salt-stressed with 1 M NaCl and incubated at 20, 30 or 50 °C for 24 h. For the experiment with stepwise osmotic stress conditions, cells were grown until late exponential phase and then salt-stressed with NaCl added in 2, 5 or 10 steps until a final concentration of 1 M for 24 h at 40 °C.

### Obtention of the low molecular weight compounds (LMW) fraction and floridoside purification

Osmotically stressed cells were harvested by centrifugation at 5000×*g* for 5 min, washed twice with ultra-pure water and freeze-dried. The dry cell pellet was mixed with 20 mL of 80 % ethanol and low molecular weight compounds were extracted from the cells by two rounds of 15 min stirring plus 15 min incubation in an ultrasonic bath (Elma) at room temperature, followed by a final incubation in a waterbath at 70 °C for 5 min Cell debris was removed by centrifugation at 10,000×*g* for 10 min and the supernatant was mixed with one volume of ultra-pure water and 0.5 volumes of chloroform. After separation of the two phases by centrifugation at 10,000×*g* for 10 min, the upper (hydroalcoholic) phase was transferred to a new tube and mixed with ionic resin Amberlite MB20 (DOW) overnight. The supernatant was concentrated under vacuum on a rotary evaporator and freeze-dried. The dry residue was resuspended in 1 mL of ultra-pure water. This was denominated the LMW fraction. Floridoside was purified from this fraction by preparative thin layer chromatography (TLC) on silica gel 60 plates (Merck-Millipore) using isopropanol:ethylacetate:water (3:1:1 by volume) as mobile phase. The identity and purity of floridoside was confirmed by ^1^H-NMR analysis. The sample was dissolved in 600 µL of deuterium oxide (D_2_O, 99.9 %_atom_, Sigma-Aldrich), freeze-dried and exchanged in the same solvent one more time. ^1^H-NMR spectra were recorded on a Varian 500 spectrometer (NMR centre, University of Groningen, The Netherlands) at a probe temperature of 25 °C. Acetone (δ ^1^H 2.225 ppm in D_2_O) was used as internal reference for chemical shift assignment and data were analysed using MestReNova 9.1.0 (Mestrelab Research S.L).

### Floridoside quantification

Floridoside fractions were analysed by high pH anion exchange chromatography coupled with pulsed amperometric detection (HPAEC-PAD) on a ICS3000 workstation equipped with a CarboPac PA-1 column (2 × 250 mm) and a ICS3000 ED detector (Dionex) using isocratic elution in 50 mM NaOH. A standard series of purified floridoside in a concentration range of 5–500 µM was used to construct a calibration curve for quantification. Floidoside yields were expressed relative to the dry biomass.

### Glycogen extraction and quantification

Glycogen was extracted from *G. sulphuraria* cells as previously described (Martinez-Garcia et al. 2016) and was quantified relative to the dry biomass. For the comparison of glycogen content at different growth phases, all cell suspensions were prepared for disruption at the same concentration of 50 mg of dry cells/mL water.

## Results

**Fig. 1:**
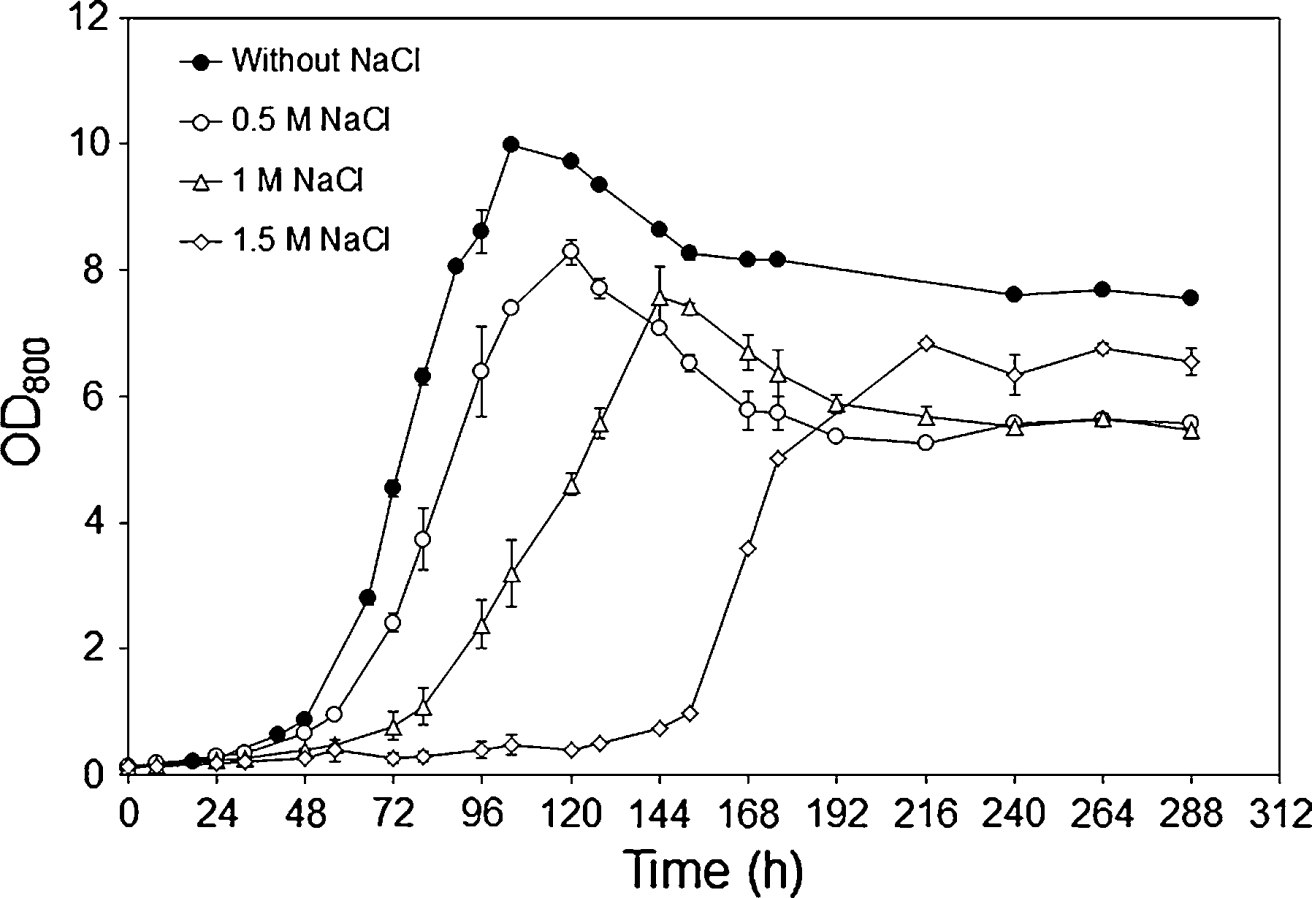
Growth curves of *G. sulphuraria* in medium containing 1 % glycerol and different NaCl concentrations at pH 2 and 40 °C in complete darkness

*Galdieria sulphuraria* was grown heterotrophically in medium containing 1 % glycerol and different NaCl concentrations and cell growth was monitored by measuring OD values at 800 nm. NaCl had an effect on both the duration of the lag phase prior exponential growth and the maximal OD value reached by the culture (Fig. 1). In cultures containing 0.5 and 1 M NaCl, the lag phase had a similar duration (48–56 h) than that of the culture without salt. Cultures containing 1.5 M NaCl showed a remarkably longer lag phase when compared to the others (144 h), after which cells could still grow exponentially. The maximum OD values were affected by NaCl in a concentration-dependent manner. Nonetheless, all salt-stressed cultures reached OD values that were close to or higher than 7.

**Fig. 2:**
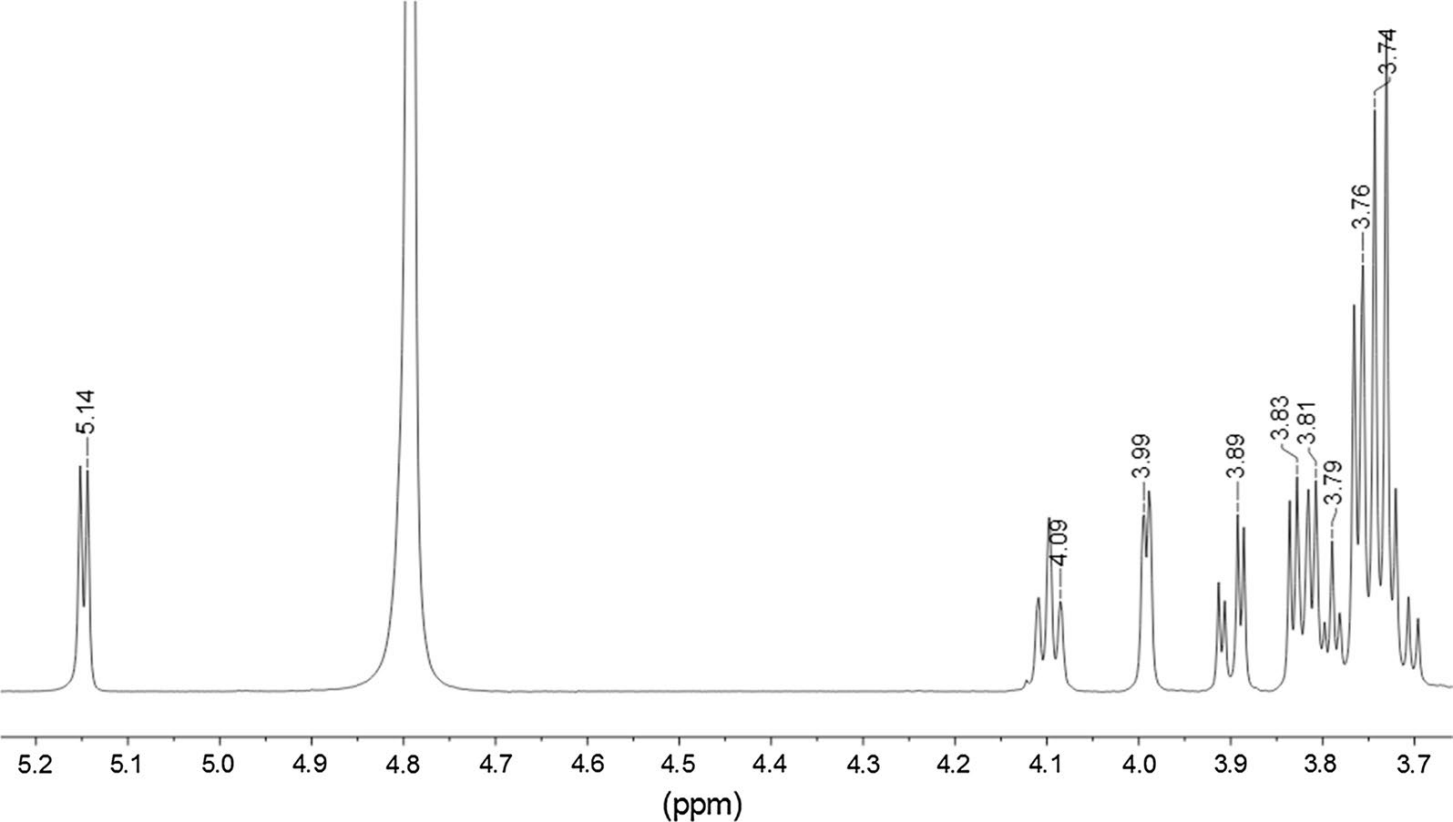
^1^H-NMR spectrum of purified floridoside from *G. sulphuraria*. The chemical shift at 4.8 ppm is the signal of residual water in the sample

In order to identify the major compatible solute in *G. sulphuraria*, the low molecular weight compounds of osmotically stressed cells were extracted with 80 % ethanol. The major constituent of this fraction was purified by preparative TLC and analysed by ^1^H-NMR (Fig. 2). The compound was identified as floridoside according to the chemical shifts reported by Simon-Colin et al. (2002). The purity of floridoside was confirmed by the absence of a signal at 4.9 ppm, characteristic of the anomeric proton in isofloridoside (1-*O*-α-d-galactopyranosylglycerol) (Bondu et al. 2007). This purified floridoside was used to prepare a calibration curve to quantify the production of the glycoside by *G. sulphuraria* under different growth and osmotic stress conditions.

Because cell growth was delayed by NaCl addition, especially at high concentrations, we decided to analyse floridoside content in cells that were osmotically stressed only after pre-growing in medium without salt and compare it to that of cells growing under osmotic stress. With this strategy, higher biomass yields could be obtained and the duration of the production process would be shortened. In order to determine the time-point of the growth curve at which the osmotic stress should be applied to obtain the highest floridoside yields, we quantified the amount of biomass, glycogen and floridoside at different growth phases (Table 1). In both late exponential and stationary growth phases, the amount of biomass (4.15 and 4.94 g dry cells/L, respectively) and the amount of glycogen accumulated by the cells (36.76 and 35.40 % of the dry biomass, respectively) were very similar. However, the amount of floridoside was 3 times higher in late exponential phase than in stationary phase. Consequently, in subsequent experiments the osmotic stress was applied once the cells had reached late exponential growth phase in medium with no salt.

**Table 1:** Biomass, glycogen and floridoside yields at different phases of *G. sulphuraria* growth in medium with 1 % glycerol and no salt

Growth phase	Biomass (g dry cells/L)	Glycogen (% dry biomass)	Floridoside (% dry biomass)
Early exponential	0.69 ± 0.09	20.07 ± 1.39	0.52 ± 0.02
Middle exponential	2.68 ± 0.51	29.39 ± 2.50	1.20 ± 0.04
Late exponential	4.15 ± 0.19	36.76 ± 2.03	1.41 ± 0.14
Stationary	4.94 ± 0.17	35.40 ± 5.79	0.47 ± 0.06

Values represent the average of three independent measurements ± standard deviation

**Fig. 3:**
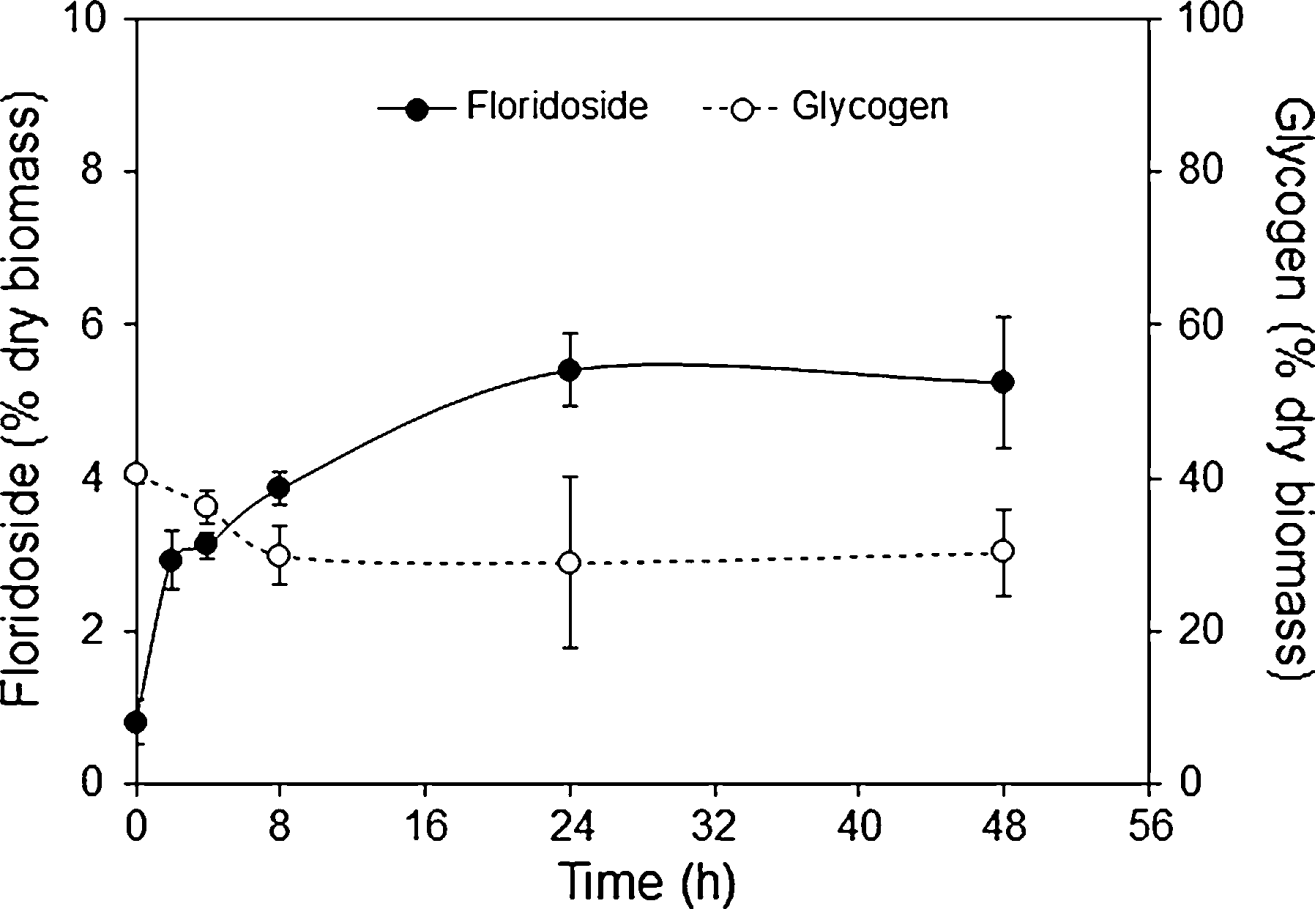
Time-course of floridoside and glycogen content in *G. sulphuraria* after addition of 1 M NaCl. Values represent the average of three independent measurements ± standard deviation

A time-course of floridoside accumulation after osmotic stress application was performed in order to identify the moment at which the amount of glycoside was maximal. Floridoside content showed almost a fivefold increase during the first 8 h after salt addition and then a more moderate increase during the following hours until reaching a maximum of 5.4 % of the dry biomass after 24 h (Fig. 3). Maintaining the osmotic stress over a longer time (48 h) did not result in higher floridoside accumulation. Glycogen content decreased slightly during the first 8 h, concomitant to the increase in floridoside during that period, and then showed variable values for the rest of the time-points, reflected by the high standard deviations obtained.

**Fig. 4:**
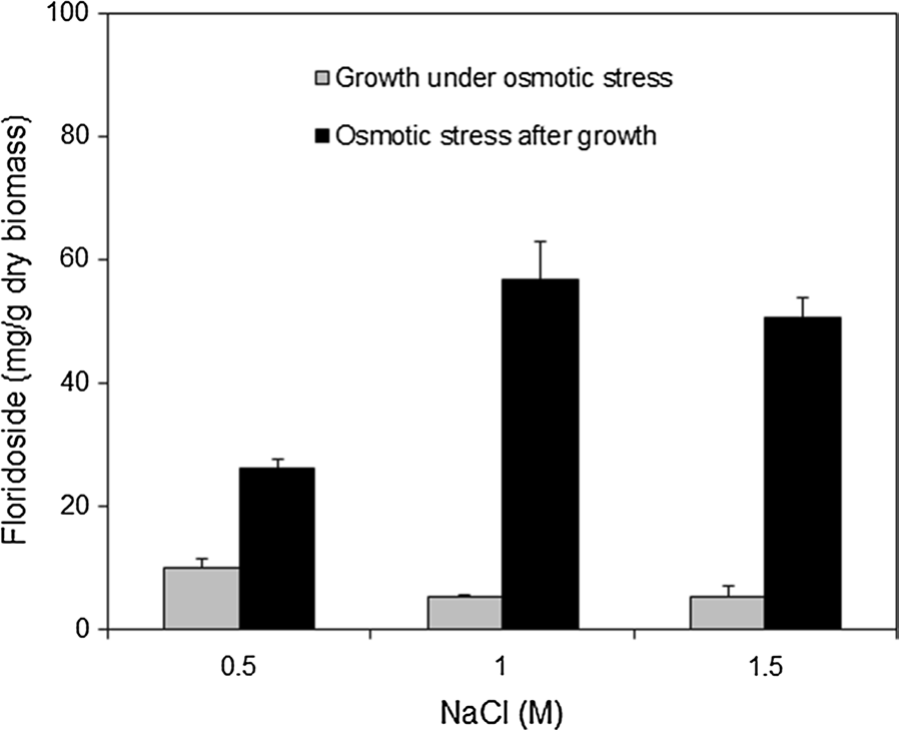
Floridoside content in *G. sulphuraria* stressed with different NaCl concentrations. Values represent the average of three independent measurements ± standard deviation

We then compared the accumulation of floridoside in late exponential phase cultures that were growing under osmotic stress on different NaCl concentrations with that of cultures pre-grown on medium with no salt and then osmotically stressed for 24 h. The latter strategy resulted in significantly increased floridoside yields, which were around 10 times higher in the case of cultures osmotically stressed with 1 and 1.5 M NaCl (Fig. 4). In cells growing under osmotic stress, floridoside accumulation did not correlate with the increase in salt concentration, since the content of glycoside was higher in cultures containing 0.5 M NaCl than in cultures with greater amounts of salt. In cells osmotically stressed after reaching late exponential growth phase, an increase in NaCl from 0.5 to 1 M resulted in a twofold increase in floridoside content, but a higher salt concentration did not yield more glycoside.

**Fig. 5:**
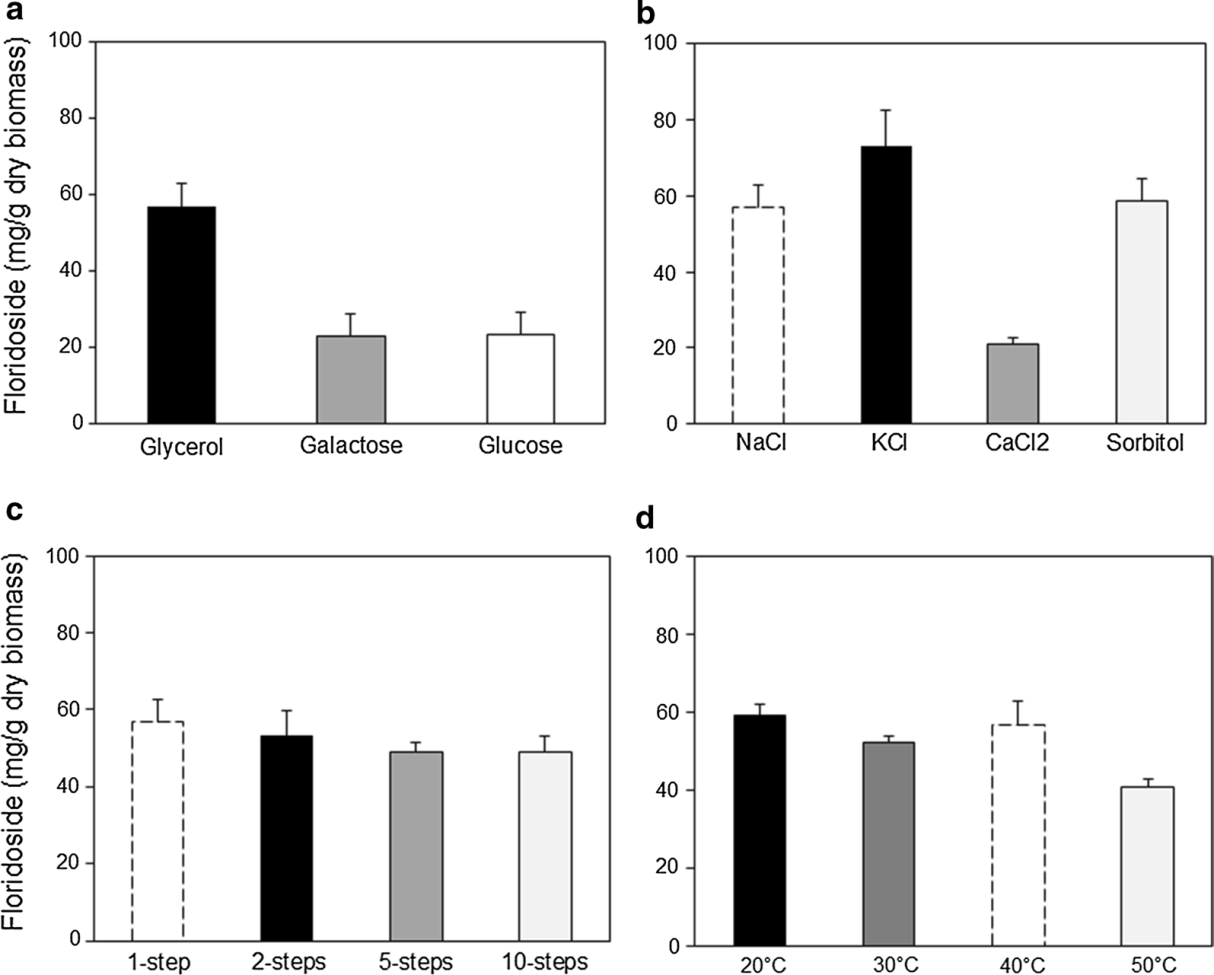
Influence of different parameters on floridoside content in *G. sulphuraria*. **a** Carbon source used for cell growth. **b** Type of compound causing the osmotic stress. **c** Stepwise addition of the osmotic agent. **d** Temperature during osmotic stress application. Columns represent the average value of three independent measurements plus standard deviation *error bars*. In all cases, cells were pre-grown on glycerol [except in (**a**)] and osmotically stressed with 1 M of solute during 24 h. For comparison purposes, the dashed border column included in (**b**–**d**) represents floridoside content in cells that were osmotically stressed with NaCl added in a single step at 40 °C

Finally, we tested the influence of different parameters on the accumulation of floridoside by *G. sulphuraria*, such as the carbon source used for cell growth, the type of compound causing the osmotic stress, the temperature of incubation and the way of applying the osmotic stress. Cells that had grown on glycerol as carbon source accumulated around 2.5 times more floridoside when subjected to osmotic stress compared to those grown on galactose or glucose (Fig. 5a). The use of sorbitol (as an example of non-ionic solute) and KCl as osmotic agents resulted in floridoside yields that were not significantly different (p > 0.05) to those obtained with NaCl (Fig. 5b). CaCl_2_ had a negative influence on floridoside accumulation, the content being at least 2.5 times lower than that obtained with the other types of solutes. The addition of the osmotic agent to the culture at once or in a stepwise manner and the temperature of incubation during the osmotic stress did not cause remarkable differences in floridoside yields (Fig. 5c, d), although cells incubated at 50 °C accumulated somewhat lower amounts of glycoside. Cultures incubated at 60 and 70 °C during osmotic stress yielded very low amounts of floridoside (data not shown).

## Discussion

Floridoside is a compatible solute known to be accumulated by marine red seaweeds (Kirst 1980; Reed et al. 1980; Ekman et al. 1995) that has attracted considerable attention for its potential antifouling and therapeutic properties (Hellio et al. 2004; Courtois et al. 2008; Kim et al. 2013; Ryu et al. 2015). However, industrial applications for floridoside have not been developed yet due to limited compound availability. A production process yielding high amounts of this glycoside would facilitate this task. With this idea in mind, we analysed floridoside production by the thermoacidophilic red microalgae *G. sulphuraria* under different conditions in order to assess its potential as industrial producer for this glycoside.

Although this microalgae species does not inhabit marine environments, it is reported to be tolerant to high concentrations of dissolved substances in the medium (Schmidt et al. 2005), including NaCl (Gross et al. 2002), and to accumulate floridoside as compatible solute (De Luca and Moretti 1983; Pade et al. 2015). Accordingly, we found that *G. sulphuraria* growth was not inhibited by NaCl, although it was considerably slowed down. Therefore, we investigated the possibility of shortening the process of floridoside production by inducing its accumulation in late-exponential cells grown in medium with no salt. With this strategy, cultures reached higher cell densities in less time and cells accumulated substantial amounts of glycogen. The storage glucan constitutes an easily accessible intracellular reserve of glucose from which the precursors of floridoside (UDP-galactose and glycerol-3-phosphate) can be synthesized (Hagemann 2016). Other red algae species have been reported to synthesize floridoside under hyperosmotic conditions from carbon obtained by degradation of the intracellular storage glucan rather than from newly assimilated carbon (Reed 1985; Ekman et al. 1995; Simon-Colin et al. 2004; Bondu et al. 2009). Interestingly, we observed that *G. sulphuraria* accumulated glycogen already at early stages of the growth curve, differing from other microorganisms where glycogen accumulation is triggered by macronutrient limitation (Lillie and Pringle 1980; Preiss 1984). *G. sulphuraria* also produced floridoside in quantifiable levels before being osmotically stressed, which is in accordance with the fact that this glycoside is not only a compatible solute but also a transient carbon reservoir (Li et al. 2001). This dual role of floridoside could be responsible for the dramatic differences in glycoside content between cells growing under osmotic stress and cells stressed only after reaching late exponential phase in medium with no salt. During prolonged (≥96 h) incubation under high osmotic pressure, other compounds known to be synthesized by *G. sulphuraria* under salt stress [e.g. betaine (Schönknecht et al. 2013)] could have taken over the role of compatible solute, allowing floridoside to function as carbon reservoir and to be degraded to sustain exponential cell growth. For cells that were osmotically stressed after growing first in medium with no salt, the shorter incubation time with salt (only 24 h) could result in a preferential accumulation of floridoside over other osmolytes.

Floridoside accumulation following an osmotic shock was a fast response. 70 % of the maximal floridoside yield was reached within the first 8 h, a similar rate to that observed for the marine red seaweed *Porphyra purpurea* (Reed et al. 1980). The decrease in glycogen content did not mirror completely the increase in floridoside, suggesting that cells might use glycogen as substrate for floridoside production only during the first hours of osmotic stress. Afterwards, compounds released from surrounding dying cells might have been the main source of precursors for floridoside synthesis, therefore eliminating the need to degrade glycogen. This seems plausible if we consider that *G. sulphuraria* possesses a high number of membrane sugar transporters to assimilate a wide range of substrates (Schönknecht et al. 2013).

Floridoside accumulation in *G. sulphuraria* cells osmotically stressed for 24 h was mainly dependant on the carbon source used for cell growth, with glycerol inducing the highest accumulation. All osmotic stress-causing agents, regardless of being ionic salts or a non-ionic solute, induced similar floridoside production, suggesting that floridoside synthesis in *G. sulphuraria* is not directly regulated by specific ions but is dependent on the external osmotic pressure, as Reed et al. (1980) described for the red macroalga *P. purpurea.* The temperature independence of floridoside accumulation would represent an advantage when considering *G. sulphuraria* as potential industrial producer for floridoside because the osmotic stress step could be performed without heat supply, thereby reducing process costs.

In conclusion, in this study we have described the culture conditions that promote the highest floridoside accumulation in *G. sulphuraria.* Further optimization of the cultivation conditions and the extraction procedure for increased biomass and floridoside yields could turn *G. sulphuraria* into an efficient industrial producer of floridoside, a promising antifouling and therapeutic compound.
